# Fibulin-5 Regulates Angiopoietin-1/Tie-2 Receptor Signaling in Endothelial Cells

**DOI:** 10.1371/journal.pone.0156994

**Published:** 2016-06-15

**Authors:** Wilson Chan, Hodan Ismail, Dominique Mayaki, Veronica Sanchez, Kerstin Tiedemann, Elaine C. Davis, Sabah N. A. Hussain

**Affiliations:** 1 Department of Anatomy and Cell Biology, McGill University, Montréal, QC, Canada; 2 Translational Research in Respiratory Diseases, McGill University Health Centre, and Meakins-Christie Laboratories, Department of Medicine, McGill University, Montréal, QC, Canada; 3 Faculty of Dentistry, McGill University and Shriners Hospital for Children, Montréal, QC, Canada; Osaka University, JAPAN

## Abstract

**Background:**

Fibulin-5 is an extracellular matrix glycoprotein that plays critical roles in vasculogenesis and embryonic development. Deletion of Fibulin-5 in mice results in enhanced skin vascularization and upregulation of the angiogenesis factor angiopoietin-1 (Ang-1), suggesting that Fibulin-5 functions as an angiogenesis inhibitor. In this study, we investigate the inhibitory effects of Fibulin-5 on Ang-1/TIE-2 receptor pathway signaling and cell survival in human endothelial cells.

**Methodology/Principal Findings:**

Recombinant wild-type and RGE-mutant Fibulin-5 proteins were generated through stable transfection of HEK293 and CHO cells, respectively. *In vitro* solid phase binding assays using pure proteins revealed that wild-type Fibulin-5 does not bind to Ang-1 or TIE-2 proteins but strongly binds to heparin. Binding assays using human umbilical vein endothelial cells (HUVECs) indicated that wild-type Fibulin-5 strongly binds to cells but RGE-mutant Fibulin-5, which is incapable of binding to integrins, does not. Pre-incubation of HUVECs for 1 hr with Fibulin-5 significantly increased caspase 3/7 activity, ERK1/2 phosphorylation, and expressions of the transcription factor early growth response 1 (EGR1) and the dual-specificity phosphatase 5 (DUSP5). Fibulin-5 also strongly attenuated Ang-1-induced TIE-2 and AKT phosphorylation, decreased Ang-1-induced expressions of the transcription factors Inhibitor of DNA Binding 1 (ID1) and Kruppel-like Factor 2 (KLF2), and reversed the inhibitory effect of Ang-1 on serum deprivation-induced cytotoxicity and caspase 3/7 activity.

**Conclusion/Significance:**

We conclude that Fibulin-5 strongly binds to the endothelial cell surface through heparin-sulfate proteoglycans and possibly integrins and that it exerts strong anti-angiogenic effects by reducing endothelial cell viability and interfering with the signaling pathways of the Ang-1/TIE-2 receptor axis.

## Introduction

Fibulins are a family of extracellular matrix (ECM) glycoproteins characterized by tandem arrays of calcium binding EGF-like domains and a C-terminal domain known as the fibulin-like module [1]. They are widely expressed, associated with basement membranes and elastic fibers, and play major roles in the assembly, stabilization, and organization of larger ECM proteins [2]. Fibulins are divided into general subgroups based on their size—long or short [3]. Long fibulins include Fibulin-1, -2, and -6. Short fibulins include Fibulin-3, -4, and -5. They are highly homologous to one another and have been associated with a variety of phenotypes related to elastic fiber pathologies [4]. Several studies have confirmed the importance of fibulins in tumorigenesis, vasculogenesis, and embryonic organ development [5–7].

Fibulin-5, also known as EVEC or DANCE, was first identified as a protein involved in the regulation of vascular smooth muscle cell (vSMC) transition from the quiescent to the proliferative state. It is strongly expressed in large blood vessels during development and its expression is upregulated in response to vascular injury [8]. Fibulin-5 gene deletion in mice (*Fbln5*^*-/-*^) results in aberrant elastic fiber formation, elastin aggregate formation, loose skin, emphysematous lungs, and tortuous blood vessels [9,10]. Our group has recently reported that *Fbln5*^*-/-*^ mice develop significantly more cutaneous blood vessels than do wild-type mice, with the formation of numerous small tortuous branches extending from the long thoracic artery, and significant increases in sprout formation and vascular invasion of subcutaneous PVA sponges are seen during *in vivo* angiogenesis [11].

Angiopoietins are oligmeric glycoproteins that bind to the endothelial cell-specific tyrosine kinase receptor, TIE-2. Angiopoietin-1 (Ang-1), the main ligand of TIE-2 receptors, is released by fibroblasts, vSMCs, and tumors, whereas angiopoietin-2 (Ang-2) is primarily released by endothelial cells [12]. Ang-1 and Ang-2 bind TIE-2 receptors with equal affinity, although Ang-2 elicits only weak phosphorylation of TIE-2 receptors and can competitively inhibit Ang-1-induced TIE-2 phosphorylation and other biological actions [12,13]. Ang-1 promotes endothelial cell survival by inhibiting apoptosis [14]. It also functions as a chemoattractant to promote endothelial cell migration [15] and as an inducer of sprouting and differentiation into tube-like structures in 2D and 3D matrices [16]. In a previous study, our group described a 30-fold increase in Ang-1 mRNA expression in cultured *Fbln5*^*-/-*^ vSMCs and suggested that Fibulin-5 may function as an anti-angiogenic factor by downregulating the expression of vascular Ang-1[11]. In this study, we address the possibility that Fibulin-5 may not only inhibit the expression of Ang-1 in endothelial cells, but may also interfere with the signaling mechanisms of Ang-1 and TIE-2 receptors and oppose the protective effects of the Ang-1/TIE-2 axis in the vasculature. To test this, we generated recombinant Fibulin-5 and assessed direct and indirect interactions between Fibulin-5, Ang-1, and TIE-2 receptors. We also examined the influence of Fibulin-5 on signaling pathways, gene expression, and regulation of endothelial cell survival by the Ang-1/TIE-2 axis.

## Materials and Methods

### Materials

Reagents used in cell culture were obtained from the Invitrogen (Burlington, ON). Recombinant human Ang-1 and Ang-2 proteins were purchased from R&D Systems (Minneapolis, MN). Both were dissolved in sterile phosphate-buffered saline (PBS). Polyclonal antibodies for phosphorylated TIE-2 (Tyr^992^)(#4221), TIE-2 receptors (#7403), phosphorylated AKT (Thre^308^)(#1308), AKT (#4685), phosphorylated ERK1/2 (Thr^202^/Tyr^204^)(#4370), ERK1/2 (#4695), phosphorylated FOXO1 (Ser^256^)(#9461), FOXO1 (#2880) and GAPDH (#2118) antibodies were obtained from Cell Signaling Inc. (Danvers, MA). Monoclonal antibody for TIE-2 receptors (clone Ab33) was obtained from Calbiochem (Darmstadt, Germany). Monoclonal antibody for Ang-1 was obtained from R&D Systems.

### Endothelial cells

Human umbilical vein endothelial cells (HUVECs) were purchased from Lonza Inc. (Mississauga, ON) and were cultured in endothelial basal medium (MCDB131) supplemented with 20% fetal bovine serum (FBS), endothelial cell growth supplements, 2 mM glutamine, heparin, penicillin, streptomycin, and amphotericin B, as previously described [17].

### Generation of recombinant full-length and RGE-mutant Fibulin-5

HEK293 and CHO cells stably expressing full-length and RGE-mutant rat Fibulin-5, respectively, were provided by Dr. H. Yangisawa (University of Texas Southwestern Medical Center, Dallas). Transfected cells were expanded and plated onto 8 triple-layer flasks in media containing 10% fetal bovine serum (FBS) (Invitrogen, Montreal, QC), 1% non-essential amino acids (NEAA) (Wisnet, St. Bruno, QC) and 1% Penicillin, Streptomycin and L-Glutamine (P/S/G) (Wisnet, St. Bruno, QC). Cells were kept under selection with either 100 μg/ml Neomycin G418 (HEK293 cells expressing full length Fibulin-5) or with 250 μg/ml Hygromycin B (CHO cells expressing RGE-mutant Fibulin-5). HEK 293 cells were incubated with Dulbecco’s Modified Eagle’s Medium (Invitrogen) and CHO cells were incubated in HAM’s F-12 (Wisent) media. Once confluent, each triple layer flask was washed twice with 150 mM NaCl in 20 mM Hepes buffer (pH 7.4) to remove serum before starting collections. A total of 4 L of serum-free media (SFM) with 1% NEAA, 1% P/S/G was collected in increments of 500 ml every second day for 3 weeks. Following each collection, the conditioned media was centrifuged at 6,000 g for 15 min at 4°C to remove cellular debris. The supernatant was collected and treated with 500 μl of 0.1 M PMSF to prevent protein degradation and frozen at -80°C.

For protein purification, the SFM was filtered using a 5 μm membrane (Millipore, Bedford, MA, Cat.#SMWP04700) under vacuum and concentrated to ~50 ml using Amicon stirred ultrafiltration cells (series 8000 system, Millipore) at 4°C. The concentrate was dialyzed using a Spectra/Por membrane MWCO 12–14,000 (Spectrum, Rancho Dominguez, CA, Cat.#132680) against 500 mM NaCl in 20 mM Hepes Buffer (pH 7.2) over a period of 24 hrs at 4°C. The dialyzed concentrate was centrifuged at 11,000 g for 16 min at 4°C and the supernatant was passed through a 1 ml chelating HisTrap affinity column (GE Healthcare, Baie d’Urfe QC) using an Äkta purification system (Amersham Biosciences, Baie d’Urfé, QC). The bound protein was eluted with a linear gradient of 500 mM Imidazole, 500 mM NaCl in 20 mM Hepes (pH 7.2). Samples (20 μl) from the fractions were separated by SDS-PAGE followed by Coomassie Blue staining to determine the presence of recombinant Fibulin-5. Fractions containing pure recombinant protein were then pooled and dialyzed using a Spectro/Por membrane MWCO 12–14,000 (Spectrum, Cat. #132676) against 2 mM EDTA in TBS (pH 7.4), twice over a period of 24 hrs at 4°C. The dialyzed pooled fractions were verified by SDS-PAGE, followed by Coomassie Blue staining and immunoblotting (see below). Protein concentration was determined using a BCA assay kit (Pierce, Rockford, IL) and a micro-ELISA plate reader (Beckman Coulter, Mississauga, Ontario). Pooled fractions were aliquoted and stored at -80°C. Recombinant full-length and RGE-mutant Fibulin-5 generated by this protocol were used for ELISAs, solid-phase binding assays and signaling experiments.

### Verification of recombinant Fibulin-5 using immunoblotting

To verify that the protein present in the fractions was purified recombinant Fibulin-5 and to detect if any degradation products were present, immunoblotting was performed. SFM media (200 μl) was collected from the triple layer flasks and precipitated in 1 ml cold acetone at -20°C for 20 min. The solution was centrifuged at 13,200 g for 10 min at 4°C. The supernatant was then discarded and the pellet was allowed to dry for 10 min under the fume hood. The pellet was then solubilized in 30 μl of 0.1 M DTT in Laemmli buffer, heated for 95°C for 5 min and then separated, transferred and probed with an IgG purified Fibulin-5 antibody as described below (see below).

### Cross-Reactivity ELISA

ELISAs were performed prior to solid-phase binding assays to ensure saturated protein coating onto the plates and to test for cross-reactivity of antibodies selective for Ang-1, TIE-2 or Fibulin-5. Full-length recombinant Fibulin-5, recombinant human Tie-2/Fc chimera (R&D Systems, Cat.# 313-TI-100) or recombinant Ang-1 (R&D, Cat. #923-AN/CF) were coated overnight at 4°C onto 96-well plates (Nalge Nunc International, Rochester, NY) in 100 μl of 4 mM CaCl_2_ in TBS at a concentration of 10 μg/ml, 4.0 μg/ml and 100 ng/ml, respectively. The following day, plates were washed for 5 min with washing buffer (20 mM Tris-HCl [pH 7.4], 150 mM NaCl, 2 mM CaCl_2_ and 0.05% Tween-20) and blocked for 1 hr at room temperature with 100 μl of 5% non-fat milk in TBS. Plates were then washed three times (5 min each) with washing buffer, and incubated for 2 hrs at room temperature with 50 μl/well of 1:50, 1:100, 1:200, 1:400, 1:800, 1:1600 and 1:3200 dilutions of primary antibodies in 2% non-fat milk in TBS. Primary antibodies used in these experiments were polyclonal rabbit IgG Fibulin-5 (generated in our laboratories) and monoclonal mouse IgG antibodies for TIE-2 (Calbiochem, Darmstadt, Germany) and Ang-1 (R&D). Plates were then washed three times with washing buffer and incubated with 50 μl/well of 1:800 dilution of secondary antibody, either peroxidase-conjugated Affini Pure goat anti-rabbit IgG or goat anti-mouse IgG (Jackson Immune Research) in 2% non-fat milk in TBS for 1.5 hr at room temperature. Plates were then washed with washing buffer and color development was performed with 100 μl/well of 1 mg/ml 5-aminosalicylic acid in 20 mM phosphate buffer (pH 6.8), including 0.045% (v/v) H_2_O_2_ for 3–5 min and stopped by adding 100 μl/well of 2 M NaOH. Color absorption measurements were taken at 490 nm using a micro-ELISA plate reader (Beckman Coulter).

### Solid-Phase binding assays

These assays were used to assess direct interactions between Ang-1, TIE-2 and Fibulin-5. Recombinant human Tie-2/Fc chimera, recombinant Ang-1, Heparin-BSA (Hep-BSA), BSA, tropoelastin (generous gift from Dr. Robert P. Mecham, Washington University School of Medicine, St. Louis, MO) and fibronectin (Sigma-Aldrich, Cat. # F1141) were coated in triplicates overnight at 4°C onto 96-well plates in 100 μl of 4 mM CaCl_2_ in TBS at a concentration of 4.0 μg/ml, 200 ng/ml, 10 μg/ml, 10 μg/ml, 10 μg/ml, and 10 μg/ml, respectively. The following day, plates were washed with for 5 min with washing buffer (20mM Tris-HCl [pH 7.4], 150 mM NaCl, 2 mM CaCl_2_ and 0.05% Tween-20) and blocked for 1 hr at room temperature with 100 μl of 5% non-fat milk in TBS. After blocking, plates were washed three times with washing buffer, and incubated for 1 hr at room temperature with 50 μl/well of 1:2 serial dilutions of recombinant Fibulin-5 in 2% non-fat milk in TBS. For competitive inhibition assays with heparin, soluble recombinant Fibulin-5 dilutions were kept at the same concentration of 20 μg/ml in 2% non-fat milk in TBS, however, they were pre-mixed with increasing amounts of soluble heparin. Plates were then washed three times with washing buffer and incubated with 50 μl/well of 1:1,000 dilution of Fibulin-5 antibody in 2% non-fat milk in TBS for 1.5 hr at room temperature. Plates were then washed with washing buffer and incubated with secondary antibody peroxidase-conjugated Affini Pure goat anti-rabbit IgG (Jackson Immune Research) in 2% non-fat milk in TBS for 1.5 hr at room temperature. Plates were again washed with washing buffer and color development was performed with 100 μl/well of 1 mg/ml 5-aminosalicylic acid in 20 mM phosphate buffer (pH 6.8), including 0.045% (v/v) H_2_O_2_ for 3–5 min and stopped by adding 100 μl/well of 2 M NaOH. Color absorption measurements were taken at 490 nm using a micro-ELISA plate reader (Beckman Coulter). In additional experiments, we repeated this protocol by coating 96-well plates with recombinant TIE-2/FC chimera or Fibulin-5 at concentrations of 4.0 μg/ml and 20 μg/ml, respectively. After washing, plates were incubated for 1 hr at room temperature with 50 μl/well of 1:2 serial dilutions of recombinant Ang-1 protein in 2% non-fat milk in TBS followed by incubation with primary Ang-1 antibody and secondary antibody as described above.

### HUVEC binding assays

Recombinant full-length and RGE-mutant Fibulin-5 and fibronectin were coated in triplicates overnight at 4°C onto 4-well chamber slides (Nalge Nunc International) in 500 μl of 4 mM CaCl_2_ in TBS, all at a concentration of 10 μg/ml. Chambers were then washed with washing buffer (20 mM Tris HCl [pH7.4], 159 mM NaCl, 2 mM CaCl_2_ and 0.05% Tween-20) and blocked for 1 hr at room temperature with 500 μl of 5% BSA in PBS. Chambers were then washed with washing buffer followed by Hepes wash buffer (20 mM Hepes, 150 mM NaCl, 2 mM CaCl_2_ [7.4]). HUVECs were trypsinized, incubated with 20%FBS medium and centrifuged at 1.1g for 6 min at room temperature. Cells were then re-suspended at a density of 150,000 cells/ml in adhesion buffer (1% BSA, 2.2 mM MgCl_2_, 0.2 mM MnCl_2_ and 10 mM Hepes in Hank’s buffered salt solution [pH 7.4])(Invitrogen). A cell suspension (500 μl/well) was placed onto each chamber and cells were allowed to bind for 1 hr in a 37°C incubator. Unbound cells were removed and the wells were washed four times with PBS and fixed by the addition of 5% paraformaldehyde for 30 min at room temperature. Cells were then rinsed twice with 1% BSA and 0.1% saponin in PBS (PBS-BS) and stained in the dark for 3 min with DAPI (Invitrogen). Cells were finally washed twice with PBS in the dark and coverslips were mounted onto slides using Vectashield mounting medium (Vector Laboratories, Burlingame, CA) and sealed with ordinary clear nail polish. Chambers were visualized with Axioskop 2 fluorescence microscope (Zeiss Inc.) and digital images (6 random fields per well chamber) were obtained with a digital camera (Axiocam, Zeiss Inc.). Images were imported into NIH ImageJ software and converted into a binary version to estimate cell number per field which was calculated using Origin software (OriginLab Corporation, Northampton, MA).

### Effects of Fibulin-5 on Ang-1/Tie-2 signaling

HUVECs were grown to ~ 80% confluency in complete medium (MCDB131 supplemented with 20% FBS and endothelial cell growth supplements). Cells were then incubated in endothelial cell basal medium lacking FBS or growth supplements. After 6 hr, HUVECs were incubated for 1 hr with PBS (control treatment) or recombinant wild-type or RGE-mutant Fibulin-5 (90 μg/ml). Cells were exposed for 10 min to PBS (control) or Ang-1 (300 ng/ml). Medium was removed and the cells were lysed in a lysis buffer. Cell lysates were assayed for phosphorylated and total levels of TIE-2 receptors, AKT, ERK1/2 and FOXO1 transcription factor using immunoblotting (see below). The rationale for using Fibulin-5 proteins at a concentration of 90 μg/ml is based on pilot experiments which revealed that incubation of HUVECs with these proteins at concentrations ranging from 60 to 120 μg/ml triggers significant changes in basal AKT and ERK1/2 phosphorylation levels (see below). In addition, we chose to stimulate HUVECs with Ang-1 for 10 min at 300 ng./ml is based on numerous studies documenting that this concentration of Ang-1 triggers within 5 to 15 min significant activation of TIE-2 receptors and downstream signaling pathways [18–21].

### Effects of Fibulin-5 and Ang-1 on endothelial cell survival and apoptosis

To evaluate the influence of Fibulin-5 on the anti-apoptotic properties of Ang-1, HUVECs were seeded into 96-well plates and maintained for 12 hr in full medium. Culture medium was replaced with basal MCDB131 culture medium (0% FBS) containing PBS (control condition), wild-type Fibulin-5 (90 μg/ml), Ang-1 (300 ng/ml), or a combination of Ang-1 and Fibulin-5. Cell cytotoxicity and caspase 3/7 activity were measured 36 hr later using a CytoTox-Fluor™ Cytotoxicity Assay and a Caspase-Glo™ 3/7 Assay, respectively, according to the manufacturer’s instructions (Promega Inc. Madison, WI). Results are expressed as percent of values measured in HUVECs cultured in full medium (20% FBS).

### Effects of Fibulin-5 on Ang-1-induced gene expression

Confluent HUVECs were maintained in basal MCDB131 culture medium for 6 hr. Cells were exposed for 1 hr to PBS, Fibulin-5 (120 μg/ml), Ang-1 (300 ng/ml), or a combination of Ang-1 and Fibulin-5. Cells were then harvested and total RNA was extracted using a Qiagen RNeasy Mini Kit, following the manufacturer’s instructions. Two μg of RNA were reverse transcribed using SuperScript II RNase H-Reverse Transcriptase (Invitrogen, Carlsbad, CA). Real-time PCR (qPCR) was performed using a Prism 7000 Sequence Detection System (Applied Biosystems, Foster City, CA) with specific primers designed to detect three transcription factors that are rapidly induced by Ang-1, namely, early growth response 1 (EGR1), kruppel-like factor 2 (KLF2), and inhibitor of DNA binding 1 (ID1) (S1 Table), and the expression of dual-specificity phosphatase 5 (DUSP5), which we recently reported as being induced by Ang-1 in endothelial cells [17,22]. Glyceraldehyde-3-phosphate dehydrogenase (GAPDH) was used as a control gene. Results were analyzed using the comparative threshold cycle (C_T_) and relative expression at a given time point was calculated as 2^-ΔΔCT^. All qPCR experiments were performed in triplicate.

### Immunoblotting

Crude HUVEC homogenates (50 μg/sample) were mixed with SDS sample buffer, boiled for 5 min at 95°C, loaded onto 8 or 10% tris-glycine sodium dodecylsulfate polyacrylamide gels, and separated by electrophoresis. Proteins were transferred by electrophoresis to polyvinylidene difluoride (PVDF) membranes then blocked with 1% BSA or milk for 1 hr at room temperature. PVDF membranes were incubated overnight at 4°C with primary antibodies followed by peroxidase (HRP)-conjugated secondary antibodies. Specific proteins were detected with an enhanced chemiluminescence (ECL) kit (Millipore, Billerica, MA). Equal loading of proteins was confirmed by stripping membranes and re-probing with β-TUBULIN antibody. Blots were scanned with an imaging densitometer and optical densities (OD) of protein bands were quantified using Image-Pro® Plus software (Media Cybernetics, Carlsbad, CA).

### Data Analysis

Data are expressed as means ± standard errors of the mean (SEM). Statistical significance was determined by one-way ANOVA with Newman-Keuls *post hoc* test. A p value of 0.05 or less was considered statistically significant.

## Results

### Generation of recombinant wild-type and RGE-mutant Fibulin-5

Secreted recombinant wild-type and RGE-mutant Fibulin-5 were purified from serum free media of stably transfected HEK293 and CHO cells, respectively. Both express a histidine tag therefore a HisTrap column was used to achieve high micromolar affinity purification of these proteins. Protein elution was performed with an imidazole gradient (S1 Fig). To identify those fractions containing Fibulin-5, several methods were employed using samples from the various fractions, including SDS-PAGE, Coomassie Blue staining, and immunoblotting with affinity purified polyclonal antibody raised against full length human Fibulin-5. Only a single protein band at the expected mass of wild-type Fibulin-5 was detected using Coomassie Blue staining and immunoblotting (S1 & S2 Figs). Purification and analyses of RGE-mutant Fibulin-5 produced similar results (data not shown). Pooled fractions of recombinant wild-type and RGE-mutant Fibulin-5 are listed in S2 Fig.

### Direct interactions between Ang-1, Fibulin-5, and TIE-2

We first verified the selectivity of Fibulin-5, Ang-1, and TIE-2 antibodies by performing ELISAs in which wild-type Fibulin-5, pure Ang-1, or TIE-2 was plated onto 96-well plates and probed with antibodies specific for each individual protein. Ang-1 antibody detected Ang-1 in a concentration-dependent manner but did not cross-react with Fibulin-5 (S3 Fig). Similarly, TIE-2 antibody did not bind to Fibulin-5 and Fibulin-5 antibody did not bind to TIE-2 or Ang-1 (S4 & S5 Figs).

**Fig 1:**
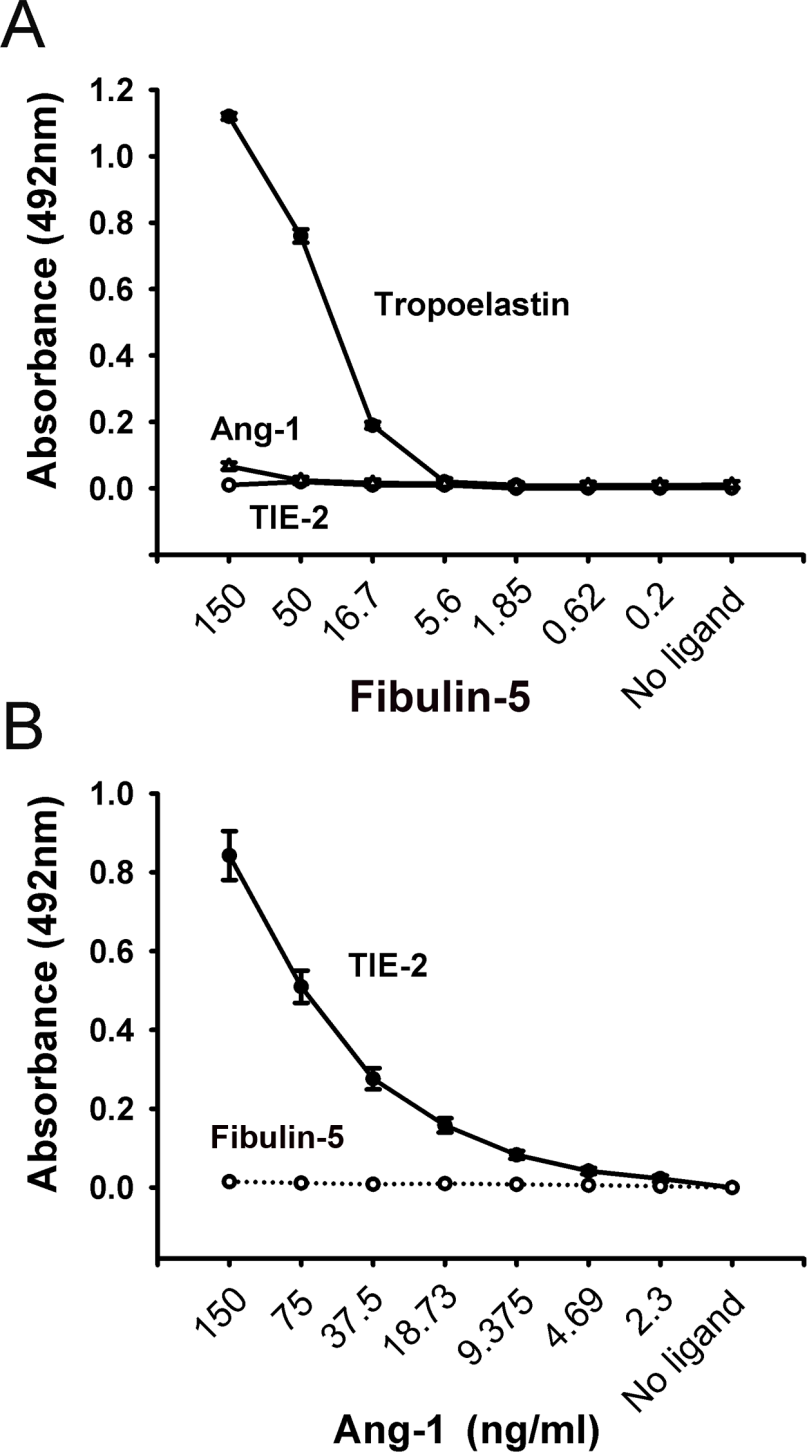
**A:** Solid-phase binding assays showing the interactions between Ang-1, TIE-2, and Fibulin-5. Tropoelastin was used as a positive control. Ang-1, TIE-2, and tropoelastin were used as immobilized ligands and Fibulin-5 as the soluble ligand. Fibulin-5 showed a strong binding for tropoelastin but no significant binding was observed to Ang-1 or TIE-2. N = 5 per group. **B:** Solid-phase binding assays showing the interactions between Ang-1, TIE-2, and Fibulin-5. TIE-2 and Fibulin-5 were used as immobilized ligands and Ang-1 protein as the soluble ligand. Ang-1 showed a strong binding to TIE-2 but no significant binding to Fibulin-5. N = 5 per group.

*In vitro* solid-phase binding assays in which pure tropoelastin (positive control), Ang-1, or TIE-2 were used as immobilized proteins and wild-type Fibulin-5 served as a soluble ligand revealed strong binding of wild-type Fibulin-5 to tropoelastin but not to Ang-1 or TIE-2 (Fig 1A). Experiments in which TIE-2 or wild-type Fibulin-5 were used as immobilized proteins and Ang-1 was used as a soluble ligand confirmed that Ang-1 does not bind to wild-type Fibulin-5 but strongly binds to TIE-2 (Fig 1B). These results indicate that Fibulin-5 is unlikely to sequester Ang-1 in the ECM and block Ang-1 binding to TIE-2 receptors on the surface of endothelial cells.

### HUVEC binding assays

**Fig 2:**
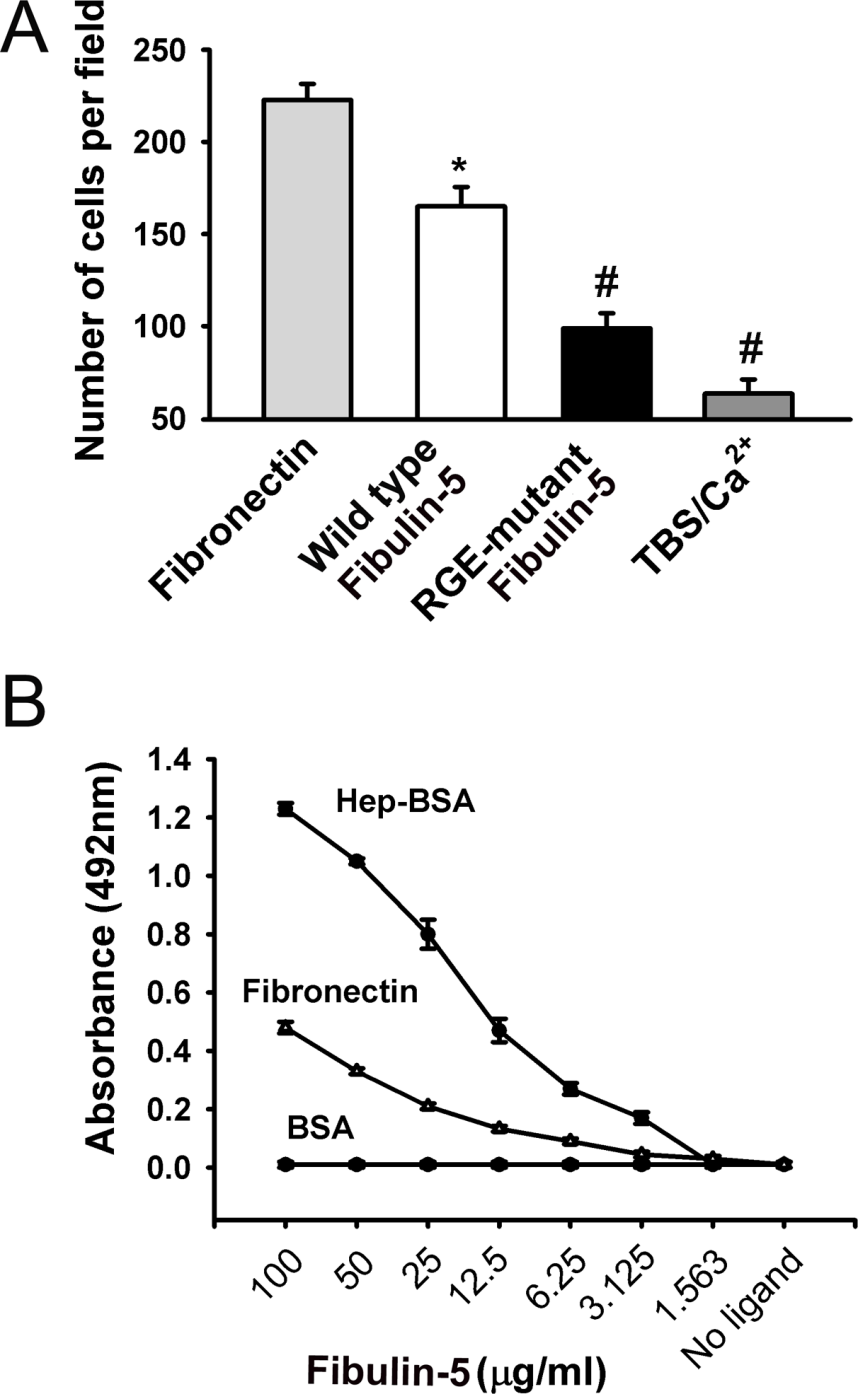
**A:** HUVEC-binding assays in which 4-chamber slides were coated with wild-type Fibulin-5, RGE-mutant Fibulin-5, TBS/Ca^2++^ buffer, or fibronectin and HUVECs were allowed to adhere to these wells for 1 hr. Values are means ± SEM of the number of HUVECs adhered to a given well. *p<0.05 compared to fibronectin. ^#^p<0.05 compared to wild-type Fibulin-5. N = 6 per group. **B:** Solid-phase binding assays showing the interaction between Fibulin-5 and heparin-BSA (Hep-BSA), fibronectin, and BSA. Hep-BSA, fibronectin, and BSA were used as immobilized ligands and Fibulin-5 as the soluble ligand. Fibulin-5 showed a binding capacity for Hep-BSA that was stronger than to the positive control fibronectin. No affinity of Fibulin-5 for BSA was detected. N = 5 per group.

Interactions between Fibulin-5 and candidate endothelial cell surface receptors such as integrins and heparan-sulfate proteoglycan were investigated by using endothelial cell and solid-phase binding assays. To test the role of integrins, RGE-mutant Fibulin-5 was generated to ablate any RGD-dependent integrin binding to Fibulin-5. Binding assays using fibronectin (positive control) and TBS buffer containing 4 mM Ca^2++^ (negative control) revealed that HUVECs strongly bind to wild-type Fibulin-5 but not to RGE-mutant Fibulin-5 (Fig 2A). These results are consistent with the involvement of integrins in the binding of Fibulin-5 to HUVECs. To assess the involvement of heparan sulfate proteoglycan in Fibulin-5 binding to endothelial cells, heparin was used, which very closely resembles heparan sulfate. BSA-conjugated heparin (Hep-BSA), BSA (negative control), and fibronectin (positive control) were used in solid-phase binding assays with increasing concentrations of wild-type Fibulin-5. Wild-type Fibulin-5 strongly binds to Hep-BSA in a concentration-dependent manner. In fact, binding is even stronger than with Fibulin-5-fibronectin binding (Fig 2B). These results confirm the involvement of heparan-sulfate proteoglycan in the binding of Fibulin-5 to HUVECs.

### Effects of Fibulin-5 on Ang-1/Tie-2 signaling

**Fig 3:**
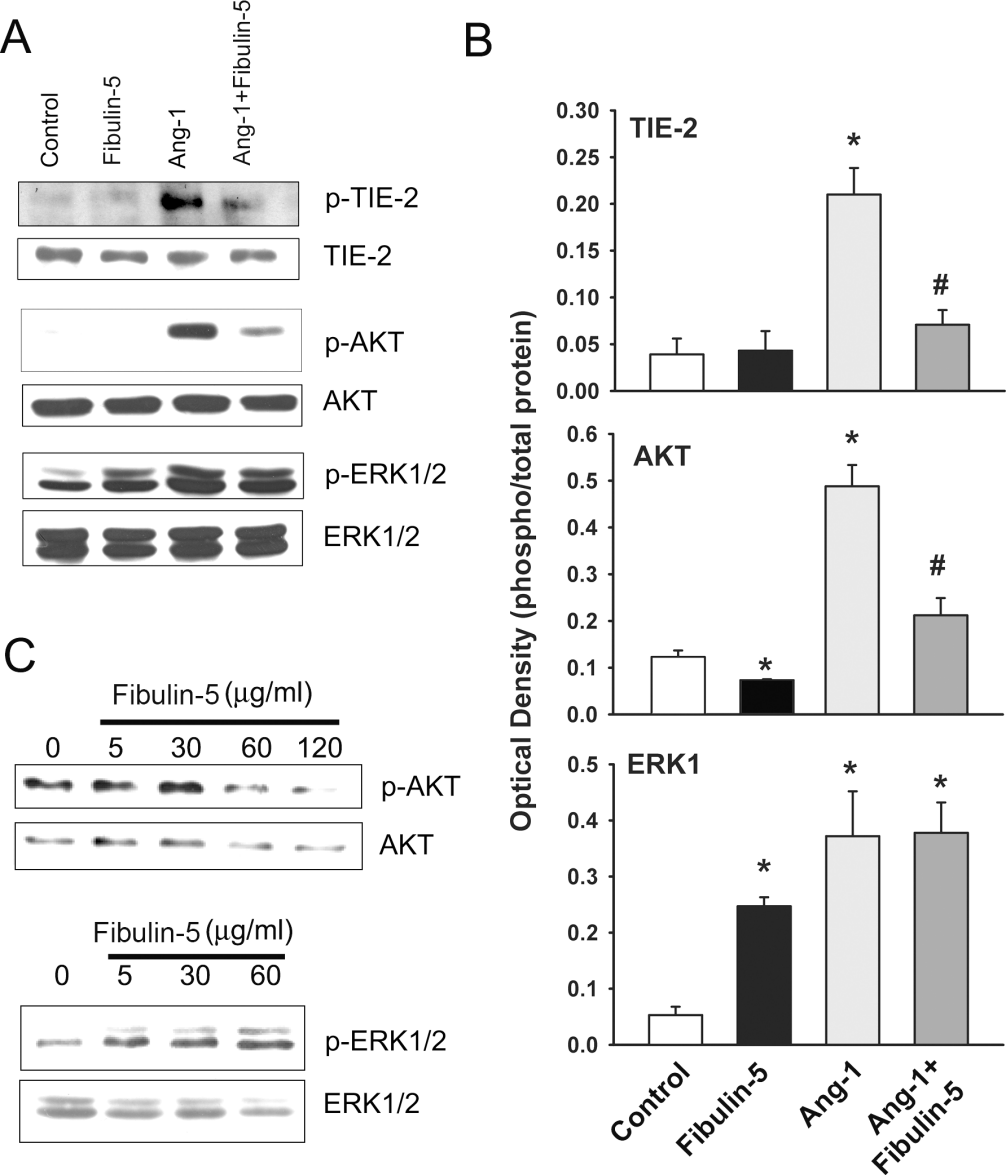
**A-B:** Representative immunoblots and optical densities of total and phosphorylated TIE-2 (Tyr^992^), AKT (Ser^473^) and ERK1/2 (Thr^202^/Tyr^204^) after 10 min of exposure to Ang-1 (300 ng/ml) in HUVECs pre-incubated for 1 hr with PBS (control) or wild-type Fibulin-5 (90 μg/ml). Values are means ± SEM. N = 4 per group. *p<0.05 compared with control. ^#^p<0.05 compared with Ang-1 alone. Note that Fibulin-5 attenuated Ang-1-induced TIE-2 phosphorylation, strongly inhibited both basal and Ang-1-induced AKT phosphorylation, and stimulated basal ERK1/2 phosphorylation. **C:** Representative immunoblots of total and phosphorylated AKT and ERK1/2 in HUVECs pre-incubated for 1 hr with PBS (0 time point) or increasing concentrations of recombinant wild-type Fibulin-5. Note that Fibulin-5 strongly inhibited AKT phosphorylation and augmented ERK1/2 phosphorylation.

Fig 3 illustrates the influence of pre-incubation for 1 hr with wild-type Fibulin-5 (90 μg/ml) on basal and Ang-1-induced TIE-2, AKT, and ERK1/2 phosphorylation in HUVECs. Wild-type Fibulin-5 exerts no effect on basal TIE-2 (Tyr^992^) phosphorylation but significantly attenuates Ang-1-induced TIE-2 phosphorylation and basal and Ang-1-induced AKT (Ser^473^) phosphorylation (Fig 3A and 3B). Basal ERK1/2 phosphorylation significantly increases but Ang-1-induced ERK1/2 phosphorylation is unaffected (Fig 3). The inhibitory effect of wild-type Fibulin-5 on basal AKT phosphorylation is concentration-dependent (Fig 3C). The stimulatory effect on basal ERK1/2 phosphorylation is present even at the 5 μg/ml concentration of wild-type Fibulin-5 (Fig 3C).

**Fig 4:**
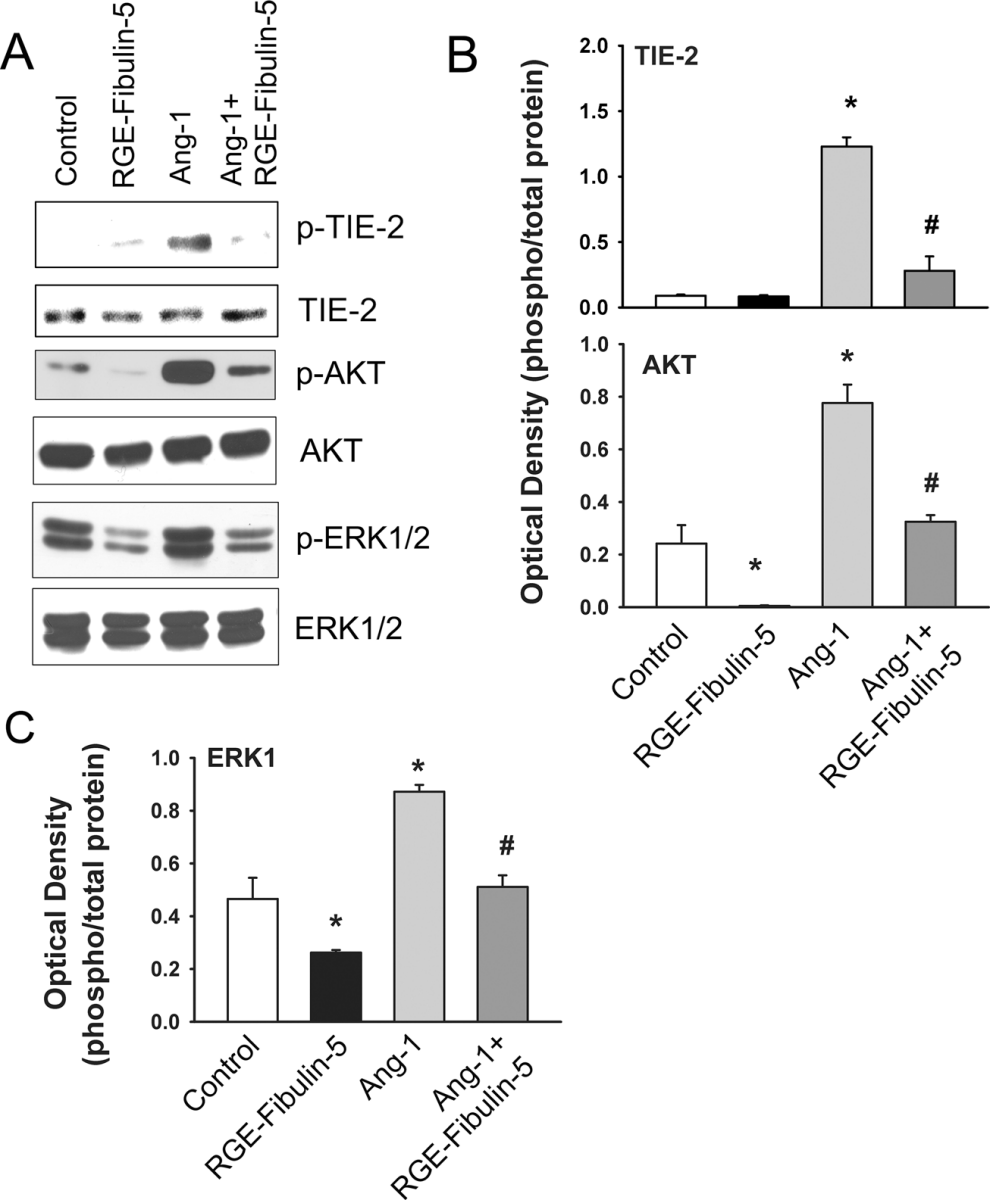
**A-C:** Representative immunoblots and optical densities of total and phosphorylated TIE-2, AKT and ERK1/2 after 10 min of exposure to Ang-1 (300 ng/ml) in HUVECs pre-incubated for 1 hr with PBS (control) or RGE-mutant Fibulin-5 (90 μg/ml). Values are means ± SEM. N = 4 per group. *p<0.05 compared with control. ^#^p<0.05 compared with Ang-1 alone. Note that RGE-mutant Fibulin-5 reduced basal and Ang-1-induced TIE-2, AKT and ERK1/2 phosphorylation.

Pre-incubation with RGE-mutant Fibulin-5 significantly attenuates both basal and Ang-1-induced TIE-2 and AKT phosphorylation (Fig 4). In contrast to wild-type Fibulin-5, RGE-mutant Fibulin-5 significantly attenuates basal ERK1/2 phosphorylation and reduces Ang-1-induced phosphorylation of ERK1/2 (Fig 4). These results are consistent with the involvement of integrins in wild-type Fibulin-5-induced ERK1/2 phosphorylation at the basal level and the absence of an inhibitory effect of wild-type Fibulin-5 on Ang-1-induced ERK1/2 phosphorylation.

**Fig 5:**
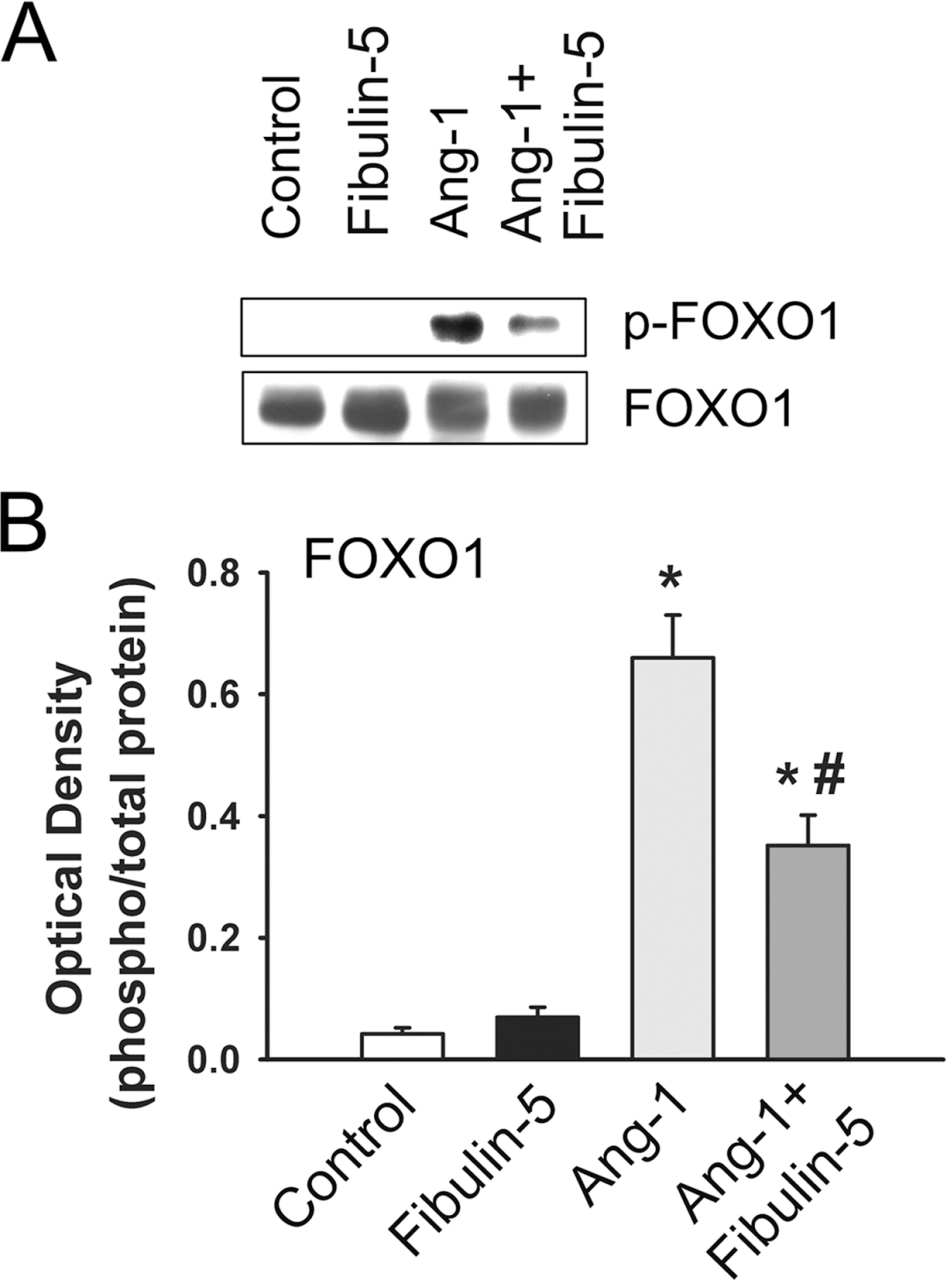
**A-B:** Representative immunoblot and optical densities of total and phosphorylated FOXO1 (Ser^256^) after 10 min of exposure to Ang-1 (300 ng/ml) in HUVECs pre-incubated for 1hr with PBS (control) or wild-type Fibulin-5 (90 μg/ml). Values are means ± SEM. N = 6 per group. *p<0.05 compared with control. ^#^p<0.05 compared to Ang-1 alone. Note that Fibulin-5 significantly attenuated Ang-1-induced FOXO1 phosphorylation.

In addition to TIE-2, AKT, and ERK1/2 phosphorylation, we evaluated whether pre-incubation with wild-type Fibulin-5 influences Ang-1-induced phosphorylation of FOXO1, an important transcription factor that is inhibited by AKT. Wild-type Fibulin-5 exerts no influence on basal FOXO1 phosphorylation (Ser^256^), but significantly attenuates Ang-1-induced increases in FOXO1 phosphorylation (Fig 5). No significant changes in total FOXO1 levels were observed in response to wild-type Fibulin-5 or Ang-1 exposure (Fig 5).

### Effect of Fibulin-5 on Ang-1-induced gene regulation

**Fig 6:**
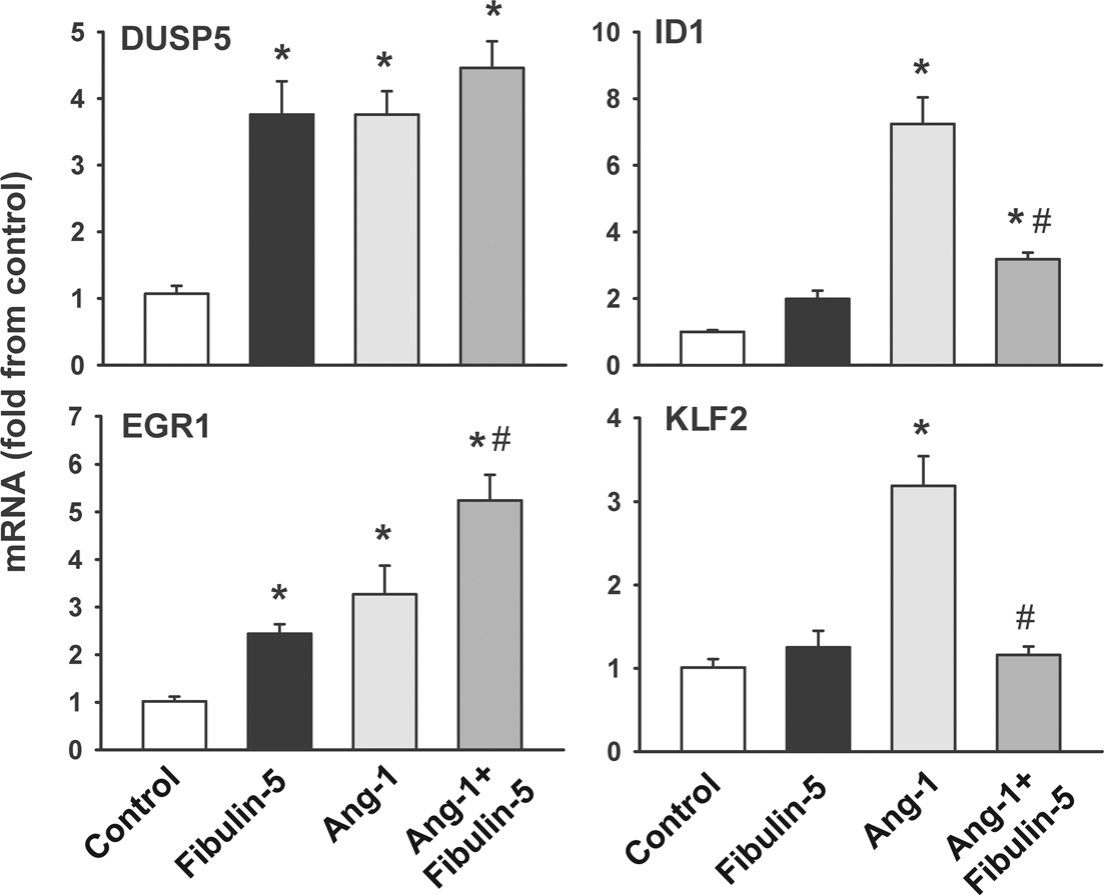
mRNA expressions of DUSP5, EGR1, ID1, and KLF2 in HUVECs pre-incubated for 1 hr with PBS (control) or wild-type Fibulin-5 (90 μg/ml) and then stimulated for 1 hr with PBS (control) or Ang-1 (300 ng/ml). *p<0.05 compared with control. ^#^p<0.05 compared with Ang-1 alone. Note that Fibulin-5 significantly increased the mRNA expressions of DUSP5 and EGR1 and significantly attenuated the expressions of Ang-1-induced ID1 and KLF2.

Pre-incubation of HUVECs for 1 hr with wild-type Fibulin-5 significantly increases the expressions of the transcription factor EGR1 and the phosphatase DUSP5 but exerts no influence on the expressions of the transcription factors ID1 and KLF2 (Fig 6). In accordance with our previous studies, exposure to Ang-1 for 1 hr significantly upregulates the mRNA expressions of DUSP5, EGR1, ID1, and KFL2 [17,23–25] (Fig 6). Wild-type Fibulin-5 significantly attenuates Ang-1-induced ID1 and KFL2 upregulation and significantly augments Ang-1-induced EGR1 upregulation (Fig 6).

### Effects of Fibulin-5 on anti-apoptotic properties of Ang-1

**Fig 7:**
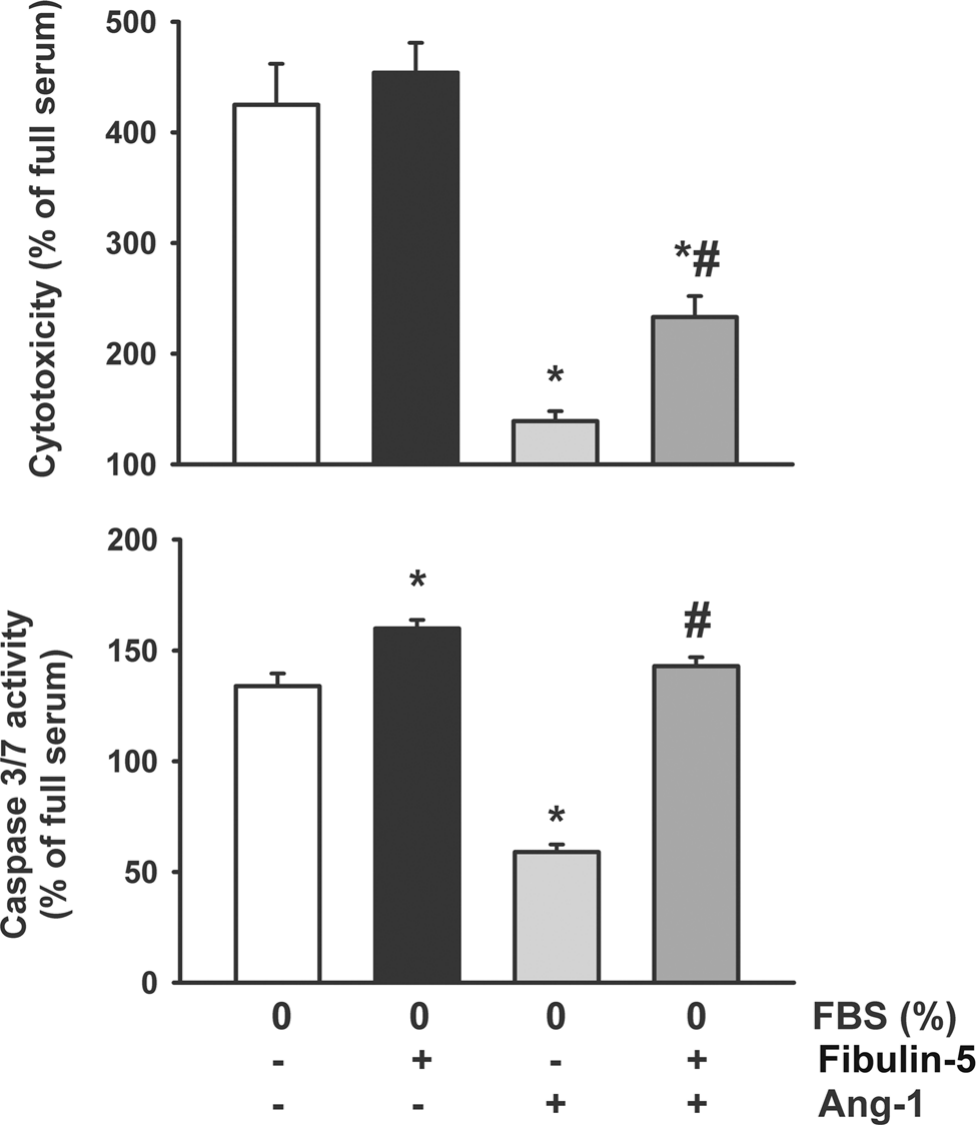
Cytotoxicity and caspase 3/7 activity in HUVECs maintained for 36 hr in media containing 0% FBS, 0% FBS + wild-type Fibulin-5, 0% FBS + Ang-1 or 0% FBS + wild-type Fibulin-5 +Ang-1. Data are expressed as % of full serum (cells maintained for 36 hr in media containing 20% FBS). *p<0.05 compared with 0% FBS. ^#^p<0.05 compared with 0% FBS + Ang-1. Note that Ang-1 significantly reduced cytotoxicity and caspase 3/7 activity and that pre-incubation with Fibulin-5 reversed that effect.

When HUVECs are maintained in basal culture medium in the absence of FBS and growth supplements, cytotoxicity and caspase 3/7 activity increases by 4- and 1.4-fold, respectively, compared to cells grown in complete medium (Fig 7). Pre-incubation for 1 hr with wild-type Fibulin-5 significantly increases caspase 3/7 activity but exerts no influence on cytotoxicity (Fig 7). The presence of Ang-1 strongly inhibits cytotoxicity and caspase 3/7 activity, effects that are either completely or partially reversed by pre-incubation with wild-type Fibulin-5 (Fig 7). This suggests that Fibulin-5 attenuates the anti-apoptotic properties of Ang-1.

## Discussion

The main findings of this study are:

1. Fibulin-5 does not directly bind to Ang-1 and TIE-2 receptors but strongly binds to heparin;
2. Endothelial cells bind to Fibulin-5 possibly through integrin-dependent mechanisms;
3. Fibulin-5 significantly increases ERK1/2 phosphorylation and upregulates DUSP5 and EGR-1 mRNA levels;
4. Fibulin-5 significantly attenuates Ang-1-induced TIE-2, AKT, and FOXO1 phosphorylation and reduces Ang-1-induced ID1 and KLF2 mRNA levels;
5. Fibulin-5 reverses the pro-survival and anti-apoptotic effects of Ang-1

Fibulin-5 is an important contributor to elastic fiber formation, as indicated by the appearance of abnormal elastic lamina formation in the aorta, and the development of pulmonary emphysema, loose skin, and tortuous vessels in *Fbln5*^*-/-*^ mice [9]. *Fbln5*^*-/-*^ mice also exhibit increased vascular sprouting, suggesting that Fibulin-5 acts as an endogenous angiogenesis inhibitor keeping vessels in a quiescent state [11]. We previously reported that the expressions of specific angiogenesis factors, such as Ang-1, are significantly elevated in vSMCs of *Fbln5*^*-/-*^ mice [11]. However, it remained unclear as to whether increased Ang-1 expression was simply an unrelated consequence of Fibublin-5 absence or whether Fibulin-5 acts as a specific inhibitor of Ang-1 expression. In the present study, we assessed whether Fibulin-5 interferes with the signaling pathways of the Ang-1/TIE-2 axis, thereby acting as an angiogenesis inhibitor by overriding the pro-angiogenic properties of this axis.

First, we characterized interactions between Fibulin-5 and the Ang-1/TIE-2 signaling pathway in terms of whether or not they are mediated through direct protein-protein interactions. Our results, obtained with solid-phase binding assays, revealed that Fibulin-5 does not bind directly to Ang-1 or TIE-2, thereby excluding the possibility that Fibulin-5 inhibits Ang-1/TIE-2 signaling by directly interfering with Ang-1 binding to TIE-2 receptors. We then evaluated how Fibulin-5 binds to the surface of endothelial cells. Contrary to what has been previously reported by Lomas *et al*. [26], we found that Fibulin-5 binds to heparin when using solid-phase binding assays. The reasons behind this discrepancy are unclear, but we speculate that differences in experimental preparation that affect Fibulin-5 protein folding might be involved. In their study, where Fibulin-5 did not bind heparin, a Biocore kinetic analysis system was used to assess the binding characteristics of a monomeric form of Fibulin-5 [26]. In our study, Fibulin-5 monomers were allowed to assemble into polymers and were then used in solid-phase binding assays.

Our observation of strong heparin binding raises the possibility that Fibulin-5 may be able to bind cell surface heparan-sulfate proteoglycans (HSPGs) such as syndecans and glypicans. Interestingly, HSPGs themselves have been implicated in anti-angiogenic activities. For instance, endostatin (an anti-angiogenic molecule) has been shown to block fibroblast growth factor- and vascular endothelial growth factor-mediated angiogenesis through its heparin-binding domain [27]. Fibulin-5 could therefore potentially act as an anti-angiogenic factor by binding cell surface HSPGs in a fashion similar to that of endostatin. Future studies are needed to identify the heparin-binding domains of Fibulin-5 and the relevance of these domains to its anti-angiogenic activity.

Previous reports have revealed that Fibulin-5 binds to HUVECs in an RGD-dependent manner [8,9]. In Chinese-hamster ovary cells, αvβ3, αvβ5, and α9β1 integrins are responsible for binding to the RGD-domain located in the N-terminal of Fibulin-5 [8,9]. In vSMCs, Fibulin-5 binds α5β1 and α4β1 integrins [26]. Our finding, that RGE-mutant Fibulin-5 weakly binds HUVECs as compared to wild-type Fibulin-5, is consistent with the notion that integrins are involved in the binding of Fibulin-5 to HUVECs [8,9]. However, our observation that mutation of the RGD motif to RGE did not completely eliminate its capacity to bind to HUVECs suggests that, in addition to the RGD motif, there may be alternate binding sites on Fibulin-5 that anchor it to the endothelial cell surface. Furthermore, it is unlikely that the RGE-mutant Fibulin-5 binding that we observed is an artifact as we verified that plate surfaces were saturated with coating protein.

Despite increasing evidence of integrin-Fibulin-5 binding on the surface of a variety of cells, the exact pathways that are activated downstream by this binding remain unclear. Lomas *et al*. [26] analyzed morphological changes in Fibulin-5-bound SMCs and reported an absence of stress fibers, an absence of focal adhesions, and diffuse actin and paxillin staining [26]. This suggests that Fibulin-5 does not activate integrin signaling pathways in these cells, a conclusion that is also based on the observation that an integrin-activating antibody rescues proliferation and migration of Fibulin-5-bound cells [26].

Our study reveals that wild-type Fibulin-5 triggers significant increases in ERK1/2 phosphorylation in endothelial cells, even at relatively low concentrations. We also observed that wild-type Fibulin-5 does not alter Ang-1-induced ERK1/2 phosphorylation. Since RGE-mutant Fibulin-5 does not elicit the same kind of increases in ERK1/2 phosphorylation that are triggered by the wild-type, it is likely that integrins are responsible for Fibulin-5-induced ERK1/2 phosphorylation since the mutant form blocks any RGD-dependent integrin binding to Fibulin-5. These findings, therefore, are consistent with the notion that Fibulin-5 is capable of influencing integrin signaling pathways, leading to significant activation of the ERK1/2 pathway in human endothelial cells. In addition to increases in ERK1/2 phosphorylation, exposing HUVECs to wild-type Fibulin-5 significantly increases DUSP5 and EGR1 mRNA levels but exerts no influence on ID1 and KLF2 expressions. Both DUSP5 and EGR1 are early response genes that are upregulated in response to several angiogenic growth factors, including VEGF and Ang-1 [17,19,28]. We have recently demonstrated that DUSP5 is significantly upregulated by the ERK1/2 pathway in HUVECs exposed to Ang-1 and that DUSP5 plays an important role in regulating ERK1/2 activity through its phosphatase activity [17]. We have also reported that the transcription of EGR1 in endothelial cells is strongly induced by the ERK1/2 pathway [19]. Accordingly, it is not surprising that DUSP5 and EGR1 upregulation coincides with activation of the ERK1/2 pathway when HUVECs are exposed to wild-type Fibulin-5. In summary, our results clearly illustrate that Fibulin-5 is capable of inducing signaling events in HUVECs and that integrins are likely to be important mediator of these events.

We have confirmed in the current study the well-established observation that Ang-1 significantly increases AKT phosphorylation in endothelial cells, a response which is mediated through TIE-2-dependent activation of the PI-3 kinase pathway [14,21,29,30]. The PI-3 kinase/AKT pathway plays a crucial role in the protection of endothelial cell survival by Ang-1 [14,21,29,30]. An important observation in our study is that wild-type Fibulin-5 significantly decreases AKT phosphorylation in a concentration-dependent manner and that Fibulin-5 strongly attenuates Ang-1-induced AKT phosphorylation and reverses the pro-survival and anti-apoptotic effects of Ang-1. We suggest that these effects can be explained by interactions between fibronectin and Fibulin-5 at the endothelial cell surface. Endothelial cells bind more strongly to fibronectin than to Fibulin-5 and there is evidence that fibronectin binds to the same types of integrins that bind to Fibulin-5 [26]. It is possible, therefore, that Fibulin-5 acts as an anti-angiogenic molecule by competing with integrin-dependent pro-angiogenic effects of fibronectin such as the modulation of VEGF-induced endothelial cell proliferation and tube formation [31]. Interactions between fibronectin and Fibulin-5 might also explain the inhibitory effects of Fibulin-5 on Ang-1/TIE-2 signaling since fibronectin has also been associated with Ang-1/TIE-2 signaling, where it sensitizes TIE-2 receptors to Ang-1 activation via α5β1 integrin [32]. α5β1 integrin is constitutively associated with TIE-2 receptors in endothelial cells. Fibronectin, by binding to α5β1, increases α5β1/TIE-2 receptor interactions, thereby triggering TIE-2 receptor clustering and potentiating their ability to bind to relatively low levels of Ang-1 [32]. Thus, it is possible that Fibulin-5 and fibronectin compete for integrin binding, resulting in either dampening or sensitization of the TIE-2 receptor response to the pro-angiogenic Ang-1 stimulus, respectively. This hypothesis is supported by the fact that aside from integrins (or possibly HSPGs), no other cell surface receptors have been shown to bind to Fibulin-5. Furthermore, Fibulin-5 does not directly bind to either TIE-2 receptors or Ang-1, thus making it unlikely that it has sequestered Ang-1 in the ECM, preventing its binding to TIE-2 receptors.

Although Ang-1-dependent endothelial cell survival is known to be mediated by the PI-3 kinase/AKT pathway, few specific targets of this pathway have been identified. Daly *et al*. [33] have identified the transcription factor FOXO1 as a target of Ang-1-induced AKT activation and reported that it is an important regulator of several genes involved in vascular destabilization and remodeling, including Sema3C, Slit2, Ang-2 (Ang-1 antagonist), and Fibulin-5 [33]. FOXO1 suppresses several pro-angiogenesis and pro-survival genes including Survivin1, which plays an important role in Ang-1-induced endothelial cell survival [14]. Thus, FOXO1 acts as a transcription factor that not only upregulates anti-angiogenesis genes, but also suppresses pro-angiogenic ones. Daly *et al*. [33] proposed that the induction of Ang-2 by FOXO1 might initiate a positive feedback loop in which Ang-2, by inhibiting the Ang-1/AKT signaling pathway, induces further increases in its own expression and in the expression of other anti-angiogenesis factors such as Slit2, Semaphorin3C, and Fibulin-5. In the current study, we confirm that Ang-1 induces significant FOXO1 phosphorylation on Ser^256^, which coincides with increased AKT phosphorylation. But more importantly, we found that pre-incubation with wild-type Fibulin-5 results in significant attenuation of Ang-1-induced FOXO1 phosphorylation. This observation raises the possibility that, like Ang-2, Fibulin-5 antagonizes the inhibitory effects of the Ang-1/AKT signaling pathway on the transcriptional activity of FOXO1, thereby promoting the expression of anti-angiogenic genes such as Ang-2, Semaphorin 3C, and Slit2 and inhibiting the expression of pro-survival genes such as Survivin-1.

In summary, we found that Fibulin-5 strongly binds to the endothelial cell surface through heparin-sulfate proteoglycans and possibly integrins. We also observed that Fibulin-5 exerts strong anti-angiogenic effects by reducing endothelial cell viability and by interfering with the signaling pathways of Ang-1/TIE-2 axis.

## Supporting Information

S1 Fig Purification of recombinant wild type Fibulin-5. **A:** Chromatogram of the purification of full-length recombinant Fibulin-5. The asterisk indicates the fractions corresponding to wild type recombinant Fibulin-5. The solid line represents the absorbance of protein, whereas the interrupted line represents the immidazole concentration gradient. Fractions of 1 ml were collected and their numbers are indicated on the x-axis. **B:** Verification of the purity of Fibulin-5 using SDS-PAGE and Coomassie Blue staining. ST: starting material. FT: Flow through.
(DOC)

S2 Fig Purification of recombinant wild type Fibulin-5. **A:** After final dialysis of the pooled fractions containing wild type Fibulin-5, SDS-PAGE and Coomassie Blue staining was performed. **B:** Immunoblotting of the pooled fractions using purified Fibulin-5 antibody to ensure no degradation fragments were present. The Fibulin-5 antibody was polyclonal raised against intact wild type Fibulin-5.
(DOC)

S3 Fig Binding of Ang-1 antibody to Ang-1 (10 ng) and wild type Fibulin-5 (1 μg) using ELISA. The x-axis represents different dilutions of Ang-1 antibody and the y-axis represents the intensity of binding of this antibody to Ang-1 or Fibulin-5. Note that Ang-1 antibody demonstrated no significant cross reactivity with Fibulin-5 and it strongly binds to Ang-1.
(DOC)

S4 Fig Binding of TIE-2 antibody to TIEe-2 and Fibulin-5 using ELISA. The x-axis represents different dilutions of TIE-2 antibody and the y-axis represents the intensity of binding of this antibody to TIE-2 (0.4μg) and wild type Fibulin-5 (1 μg). Note that TIE-2 antibody demonstrated no significant cross reactivity with Fibulin-5 protein but strongly binds to TIEe-2.
(DOC)

S5 Fig Binding of Fibulin-5 antibody to TIEe-2 and Ang-1 using ELISAs. The x-axis representdifferent dilutions of Fibulin-5 antibody and the y-axis represents the intensity of binding of this antibody to Fibulin-5 (1 μg/ml), TIE-2 (0.4μg) and Ang-1 (10 ng). Note that Fibulin-5 antibody demonstrated no significant cross reactivity with TIE-2 and Ang-1 but it strongly binds to Fibulin-5.
(DOC)

S1 Table List of primers used for Real-Time PCR experiments. (DOC)
