# Supplementary material for: Fibulin-5 Regulates Angiopoietin-1/Tie-2 Receptor Signaling in Endothelial Cells
Source: PLoS One. 2016 Jun 15;11(6):e0156994. doi: 10.1371/journal.pone.0156994 (PMC4909301; doi:10.1371/journal.pone.0156994)
Supplement: S1 Fig — A: Chromatogram of the purification of full-length recombinant Fibulin-5. The asterisk indicates the fractions corresponding to wild type recombinant Fibulin-5. The solid line represents the absorbance of protein, whereas the interrupted line represents the immidazole concentration gradient. Fractions of 1 ml were collected and their numbers are indicated on the x-axis. B: Verification of the purity of Fibulin-5 using SDS-PAGE and Coomassie Blue staining. ST: starting material. FT: Flow through. (DOC) [file pone.0156994.s001.doc]

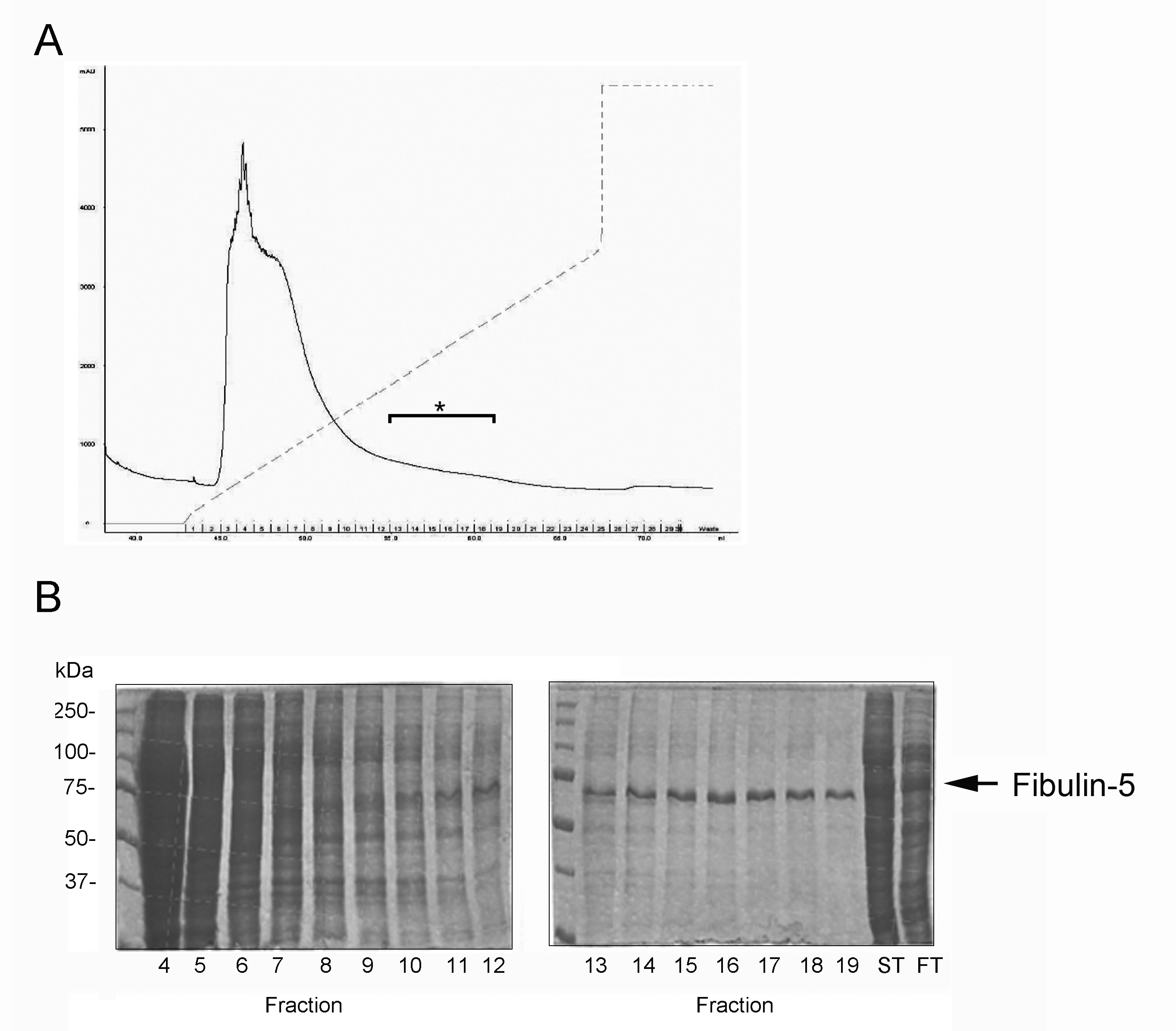


**S1 Fig.**

**Purification of recombinant wild type Fibulin-5: A:** Chromatogram of the purification of full-length recombinant Fibulin-5. The asterisk indicates the fractions corresponding to wild type recombinant Fibulin-5. The solid line represents the absorbance of protein, whereas the interrupted line represents the immidazole concentration gradient. Fractions of 1 ml were collected and their numbers are indicated on the x-axis. **B:** Verification of the purity of Fibulin-5 using SDS-PAGE and Coomassie Blue staining. ST: starting material. FT: Flow through.
