# Supplementary material for: Fibulin-5 Regulates Angiopoietin-1/Tie-2 Receptor Signaling in Endothelial Cells
Source: PLoS One. 2016 Jun 15;11(6):e0156994. doi: 10.1371/journal.pone.0156994 (PMC4909301; doi:10.1371/journal.pone.0156994)
Supplement: S2 Fig — A: After final dialysis of the pooled fractions containing wild type Fibulin-5, SDS-PAGE and Coomassie Blue staining was performed. B: Immunoblotting of the pooled fractions using purified Fibulin-5 antibody to ensure no degradation fragments were present. The Fibulin-5 antibody was polyclonal raised against intact wild type Fibulin-5. (DOC) [file pone.0156994.s002.doc]

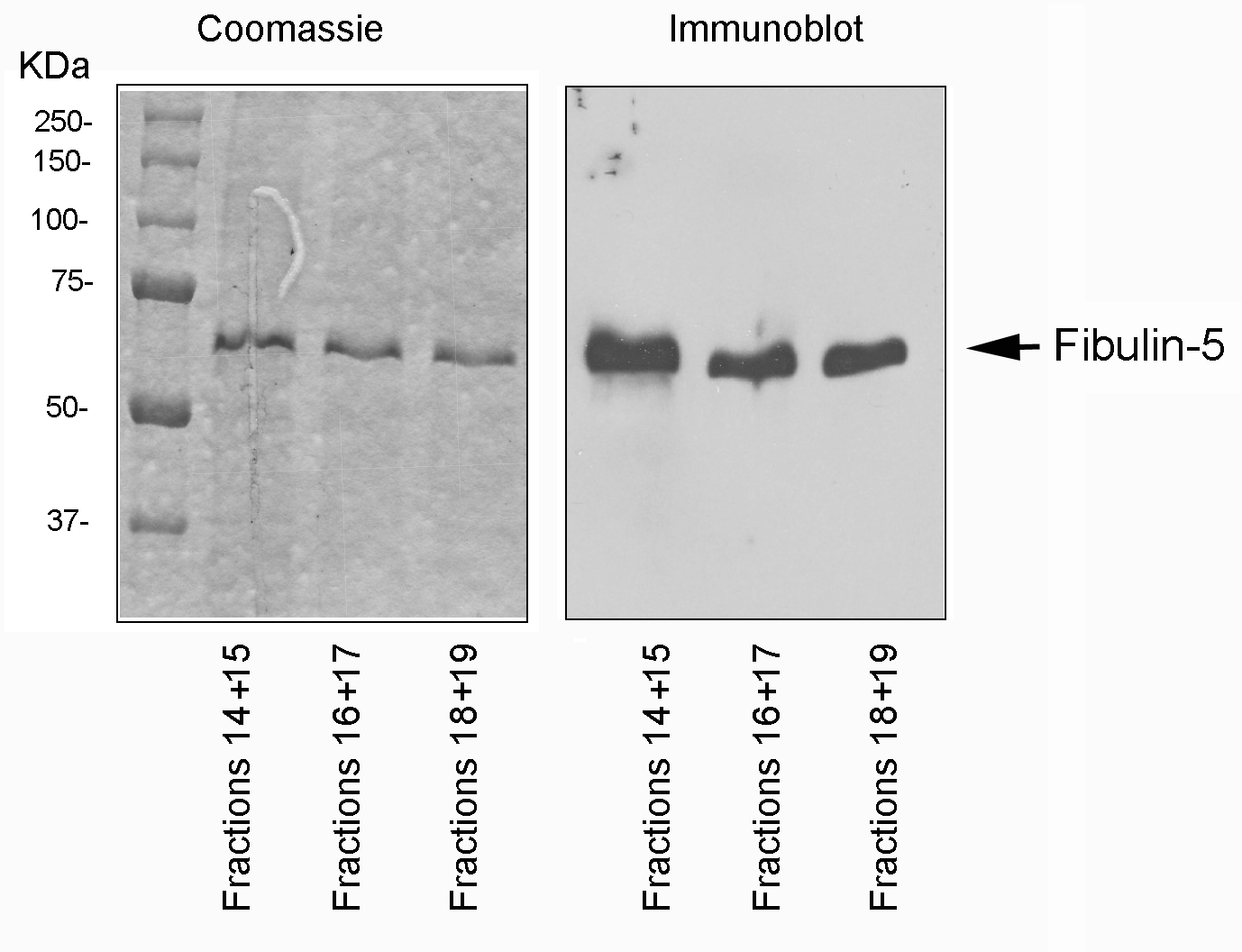


**S2 Fig.**

**Purification of recombinant wild type Fibulin-5: A:** After final dialysis of the pooled fractions containing wild type Fibulin-5, SDS-PAGE and Coomassie Blue staining was performed. **B:** Immunoblotting of the pooled fractions using purified Fibulin-5 antibody to ensure no degradation fragments were present. The Fibulin-5 antibody was polyclonal raised against intact wild type Fibulin-5.
