# Supplementary material for: Fibulin-5 Regulates Angiopoietin-1/Tie-2 Receptor Signaling in Endothelial Cells
Source: PLoS One. 2016 Jun 15;11(6):e0156994. doi: 10.1371/journal.pone.0156994 (PMC4909301; doi:10.1371/journal.pone.0156994)
Supplement: S3 Fig — The x-axis represents different dilutions of Ang-1 antibody and the y-axis represents the intensity of binding of this antibody to Ang-1 or Fibulin-5. Note that Ang-1 antibody demonstrated no significant cross reactivity with Fibulin-5 and it strongly binds to Ang-1. (DOC) [file pone.0156994.s003.doc]

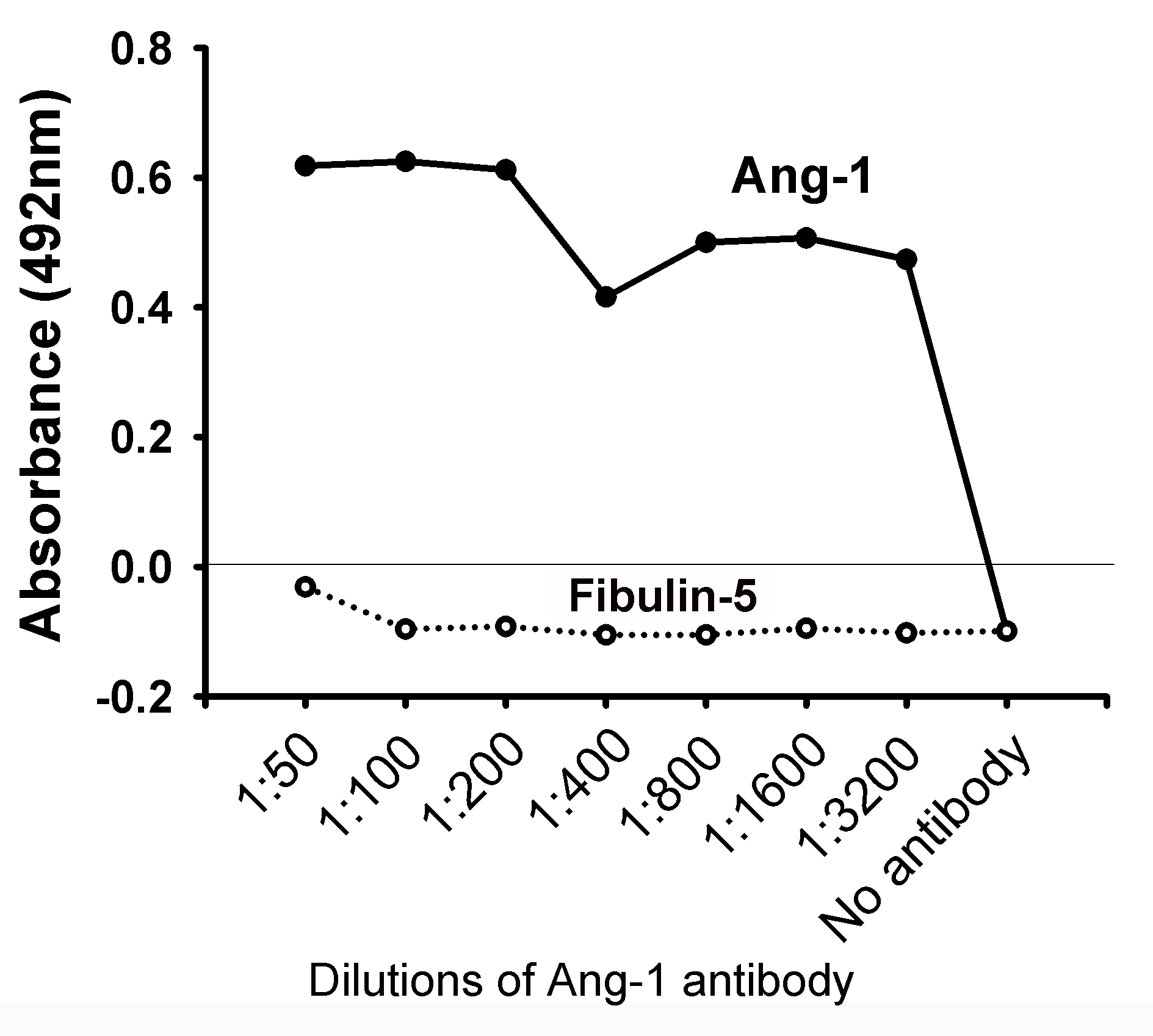


**S3 Fig.**

Binding of Ang-1 antibody to Ang-1 (10 ng) and wild type Fibulin-5 (1 μg) using ELISA. The x-axis represents different dilutions of Ang-1 antibody and the y-axis represents the intensity of binding of this antibody to Ang-1 or Fibulin-5. Note that Ang-1 antibody demonstrated no significant cross reactivity with Fibulin-5 and it strongly binds to Ang-1.
