# Supplementary material for: Fibulin-5 Regulates Angiopoietin-1/Tie-2 Receptor Signaling in Endothelial Cells
Source: PLoS One. 2016 Jun 15;11(6):e0156994. doi: 10.1371/journal.pone.0156994 (PMC4909301; doi:10.1371/journal.pone.0156994)
Supplement: S4 Fig — The x-axis represents different dilutions of TIE-2 antibody and the y-axis represents the intensity of binding of this antibody to TIE-2 (0.4μg) and wild type Fibulin-5 (1 μg). Note that TIE-2 antibody demonstrated no significant cross reactivity with Fibulin-5 protein but strongly binds to TIEe-2. (DOC) [file pone.0156994.s004.doc]

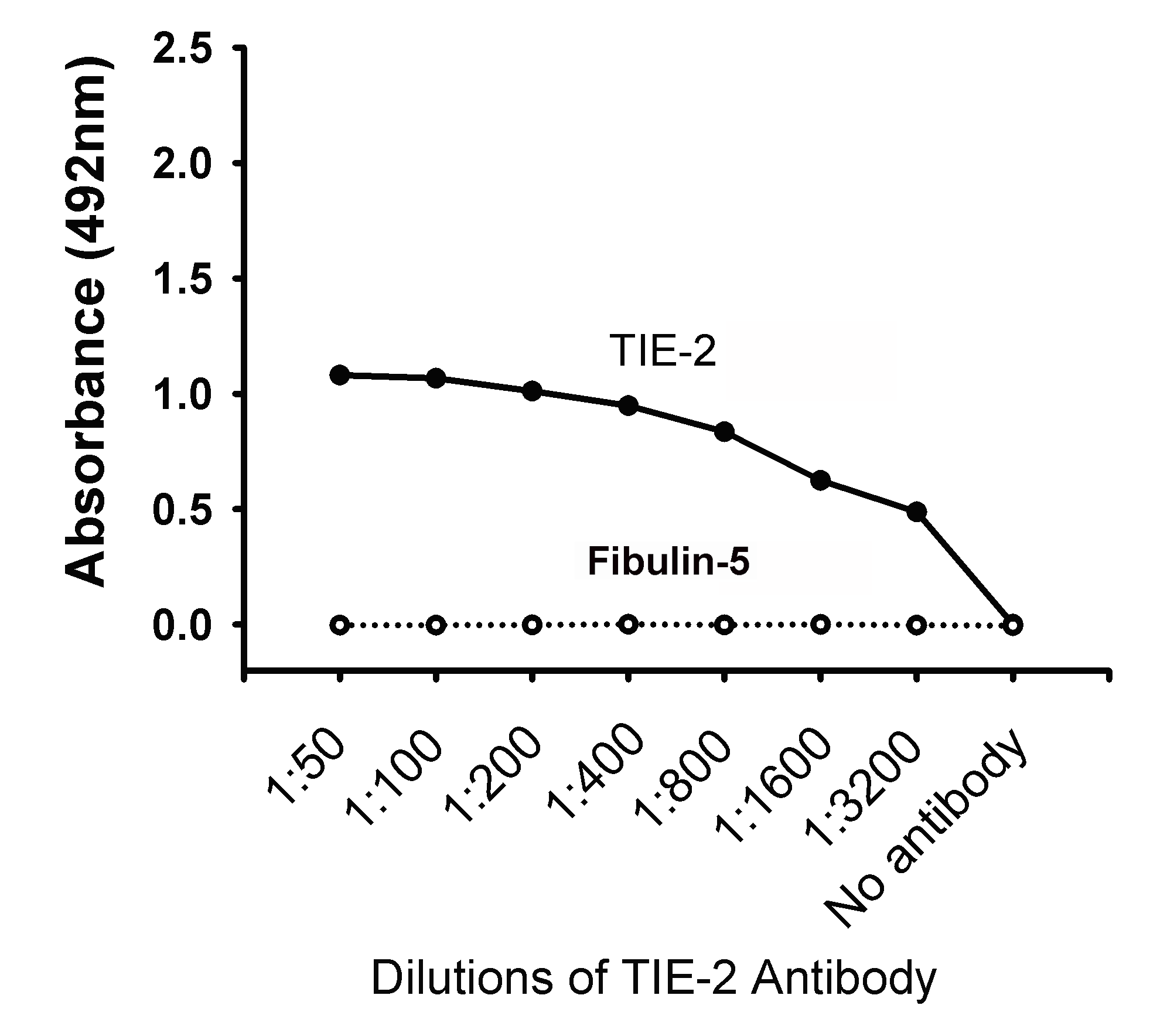


**S4 Fig.**

Binding of TIE-2 antibody to TIEe-2 and Fibulin-5 using ELISA. The x-axis represents different dilutions of TIE-2 antibody and the y-axis represents the intensity of binding of this antibody to TIE-2 (0.4μg) and wild type Fibulin-5 (1 μg). Note that TIE-2 antibody demonstrated no significant cross reactivity with Fibulin-5 protein but strongly binds to TIEe-2.
