# Supplementary material for: Fibulin-5 Regulates Angiopoietin-1/Tie-2 Receptor Signaling in Endothelial Cells
Source: PLoS One. 2016 Jun 15;11(6):e0156994. doi: 10.1371/journal.pone.0156994 (PMC4909301; doi:10.1371/journal.pone.0156994)
Supplement: S5 Fig — The x-axis representdifferent dilutions of Fibulin-5 antibody and the y-axis represents the intensity of binding of this antibody to Fibulin-5 (1 μg/ml), TIE-2 (0.4μg) and Ang-1 (10 ng). Note that Fibulin-5 antibody demonstrated no significant cross reactivity with TIE-2 and Ang-1 but it strongly binds to Fibulin-5. (DOC) [file pone.0156994.s005.doc]

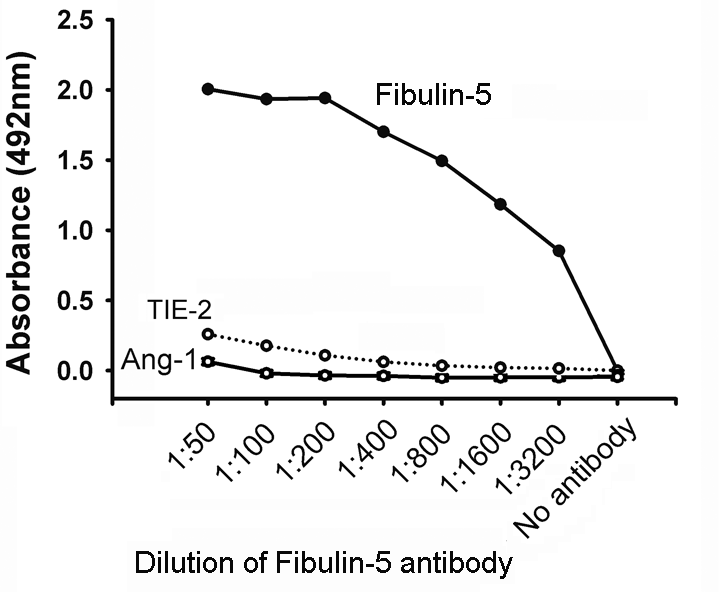


**S5 Fig**

Binding of Fibulin-5 antibody to TIEe-2 and Ang-1 using ELISAs. The x-axis represents different dilutions of Fibulin-5 antibody and the y-axis represents the intensity of binding of this antibody to Fibulin-5 (1 μg/ml), TIE-2 (0.4μg) and Ang-1 (10 ng). Note that Fibulin-5 antibody demonstrated no significant cross reactivity with TIE-2 and Ang-1 but it strongly binds to Fibulin-5.
