# Supplementary material for: Fibulin-5 Regulates Angiopoietin-1/Tie-2 Receptor Signaling in Endothelial Cells
Source: PLoS One. 2016 Jun 15;11(6):e0156994. doi: 10.1371/journal.pone.0156994 (PMC4909301; doi:10.1371/journal.pone.0156994)
Supplement: S1 Table — (DOC) [file pone.0156994.s006.doc]

**S1 Table:** List of primers used for Real-Time PCR experiments

**Gene Forward Primer (Tm) Reverse Primer (Tm) Product Size (bp)**

**DUSP5**  GGATCCCTGTGGAAGACAGC GACCATGCTCCTCCTCTGCT 221

**EGR1** CACGCCGAACACTGACATTT TAGTCGGGGATCATGGGAAC 135

**ID1** TGAGCTTGCTGGACGACATG GATGACGCGCTGTAGGATTTC 110

**KLF2** GCACGCACACAGGTGAGAAG ACCAGTCACAGTTTGGGAGGG 269

**GAPDH** AAGAAGGTGGTGAAGCAGGCG ACCAGGAAATGAGCTTGACAA 166
